# An Assessment of the Feasibility and Effectiveness of Distance Learning for Students With Severe Developmental Disabilities and High Behavioral Needs

**DOI:** 10.1007/s40617-020-00549-1

**Published:** 2021-03-01

**Authors:** Melaura Andree Erickson Tomaino, Alissa L. Greenberg, Sarah Ann Kagawa-Purohit, Sagui A. Doering, Edward Steven Miguel

**Affiliations:** 1 Port View Preparatory, 1361 Valencia Avenue, Tustin, CA 92780 USA; 2 Focused Behavioral Solutions, Berkeley, CA USA

**Keywords:** Distance learning, COVID-19, Special education, Developmental disabilities, Social validity

## Abstract

Schools across the country closed their doors during the COVID-19 pandemic. These measures impacted all students, as schools, educators, and families grappled with the realities of transitioning to distance-learning platforms. The research on distance learning is still in its early phases. However, almost no research exists on educating students with severe disabilities and high behavioral needs using this technology. Study 1 collected survey data from students’ families and their educators on the feasibility and effectiveness of distance-learning programs when working with students with severe developmental disabilities and high behavioral needs. Results indicated that parents and educators had generally neutral attitudes toward distance learning, although educators agreed that their students were obtaining educational benefits during distance learning. Study 2 further examined the effects of a transition to distance learning on students’ Individualized Education Plan (IEP) goal progress. Analyses revealed that students maintained about half of the skills addressed in their IEPs and made progress on an additional quarter of their IEP goals. Findings contribute to a much-needed literature base on distance learning and provide additional information as to the feasibility and effectiveness of distance learning with students with severe developmental disabilities and high behavioral needs. Future work is needed to determine best practices for distance learning with this population.

In the early spring of 2020, COVID-19 hit the United States. COVID-19 is an illness caused by a virus that spreads from person to person and can live in the air for over 3 hr and on some surfaces for up to 3 days (van Doremalen et al., 2020). The United States detected its first case of COVID-19 on January 21, 2020 (Centers for Disease Control and Prevention [CDC], 2020a). Soon after, several states issued shelter-in-place orders, leading to the closure of the majority of businesses and educational institutions across the United States. These orders have changed all aspects of society, including education. By April 2020, the pandemic had led to 96% of schools across the United States closing in an effort to keep students safe and healthy (MCH Strategic Data, 2020). This represents 97.91% of the entire K–12 population of 57,900,000 students (MCH Strategic Data, 2020).

These school-closure measures have impacted all students in unprecedented ways as schools, educators, and families grappled with the realities of transitioning to distance-learning platforms. Distance learning or education is defined as a method of teaching where the student and teacher are physically separated (Kentnor, 2015). It can utilize a variety of technologies, including correspondence, audio, video, computers, and the internet (Roffe, 2004). Distance education is also called “online education” and most recently utilizes computers and the internet as the delivery mechanism, with at least 80% of the course content delivered online (Allen & Seaman, 2011; Shelton & Saltsman, 2005). There are various delivery models of distance learning, including asynchronous and synchronous modalities. Asynchronous models involve a method of communication where messages are sent and received over a period of time, and include two-way communication in which there is a time delay between the message being sent and the message being received. Alternatively, synchronous modalities involve online communication between two or more people at once, but not necessarily in the same place. It is a computer-mediated exchange of information where participants are online simultaneously (Mahoney & Hall, 2020).

Although the COVID-19 pandemic was the impetus for the present study, there are other variables beyond a national crisis that may result in students transitioning to distance-learning models, including other emergencies (e.g., fire). Other examples may include students who demonstrate personalized education needs (Murphy et al., 2011), families or students who need a more self-paced curriculum (Murphy et al., 2011), or students who struggle with traditional classroom environments (Repetto et al., 2010). Specifically, for students with special needs, research has shown that the flexibility of course scheduling, the flexibility of lesson pace with the ability to take breaks, automated and immediate feedback, and the reduced pressure of having to perform in front of peers are benefits of distance education learning modalities (Marteney & Bernadowski, 2016). The National Center for Education Statistics (2020) reported that during the 2017–2018 school year, 59% of public schools offered at least one course entirely online, and 21% of public schools offered all of their courses entirely online.

Although previous numbers of students enrolled in distance learning were high, the COVID-19 pandemic has now resulted in the majority of schools across the nation transitioning from brick-and-mortar education to distance learning with almost no time to plan (MCH Strategic Data, 2020). Despite this widespread transition to distance learning, the research on distance learning is still in its early phases, and little research has assessed the effects of online lessons for elementary, middle, and high school students. Of those studies, findings remain mixed. Several studies have demonstrated equal effectiveness of online learning platforms and face-to-face instruction (Barbour & Mulcahy, 2009; Barker & Wendel, 2001). Further, others have even documented the superiority of online platforms over traditional face-to-face learning (Cavanaugh, 2001; Hart, Berger, Jacob, Lomb, and Hill, 2019). Despite such promising data, other studies document negative effects of moving to distance learning (Fitzpatrick, Berends, Ferrare, & Waddington, 2020). Even less is known about the mediating and moderating variables that impact the effectiveness of distance learning and whether there are differences in outcomes across age, race, and socioeconomic factors (Cavanaugh et al., 2004). These mixed results and limited research base make it difficult to truly understand the impact on students’ education of moving from traditional face-to-face instruction to distance-learning platforms.

Most of the research on distance learning has focused on the general education population. Fewer studies have looked at the impact of distance learning on students with special needs, let alone those with severe developmental disabilities and high behavioral needs. These students and their families are now in uncharted waters. Although some studies indicate that distance-learning programs are uniquely positioned to meet the needs of students with disabilities (Hashey & Stahl, 2014; Rose & Blomeyer, 2007), research in this area is scarce, and findings are mixed (Means et al., 2010). Heiman (2006) found that university students with learning disabilities can be successful in distance-learning programs with additional supports. However, this is not representative of the entire special education population, as participants ranged in age from 18 to 50 years and had IQ scores of 95–120. Findings therefore cannot be generalized to younger students or those with intellectual disabilities. Additional studies investigated teachers’ experiences with distance learning. For example, Marteney and Bernadowski (2016) surveyed teachers who taught an online education program for students with special education needs. In this study, the majority of the teachers reported that online education opened up access to learning activities for students with limitations, and reported improvements in student academic performance. These findings are extremely promising and encouraging; however, as with distance-learning studies performed with the general education population, research including the special education population also remains mixed. For example, Carnahan and Fulton (2013) found that students with special needs enrolled in a cyberschool performed worse than the state average for special needs students on standardized math and reading education assessments. In this study, cyberschools were defined as charter schools that were delivering instruction through computer-based formats.

The research on distance learning and students with special needs is further restrained by the limited variability of students represented in these studies. In 2012, Vasquez and Straub conducted a review of the literature on online learning for special education students. Their review identified 43 studies on this topic. Of those studies, only six were identified as empirical. A deeper dive into the six empirical studies revealed that, although most students had an individualized education plan (IEP), students in three of the six empirical studies participated in mainstream classrooms, suggesting that their developmental or behavioral limitations did not require more restrictive placements. When looking at the percentage of students with special needs enrolled in distance schools, diagnoses that often represent students with severe challenging behaviors and significant developmental delays represented a very small portion of those enrolled. For example, Carnahan and Fulton (2013) investigated distance schools in Pennsylvania. Of the 2,600 students with special needs enrolled in distance-learning schools, the majority of the students were classified as having a learning disability. Less than 10% were diagnosed with autism spectrum disorder (ASD) or intellectual disability (ID), highlighting the need for research specifically looking at those individuals who are more severely impacted.

Developmental disabilities encompass a broad range of conditions that result from cognitive and/or physical impairments and range in degree of severity (May Institute, 2020). Seventeen percent of individuals ages 3–17 years are diagnosed with a developmental disorder, the most common of which are ID, cerebral palsy, and ASD (CDC, 2020b). Further, Petek (2019) outlined requirements set forth by the Individuals With Disabilities Education Act (IDEA) and reported both ASD and ID are considered relatively severe disabilities. In addition to exhibiting delays in development, many of these individuals also demonstrate challenging behavior. Research suggests that approximately 64%–93% of individuals with ASD exhibit one or more challenging behaviors (McTiernan et al., 2011), and 10%–20% of individuals with ID demonstrate challenging behaviors (Jacobson, 1982; Kiernan & Kiernan, 1994). These students are often educated with supports and services beyond what is offered in general education classrooms and at times in different classrooms altogether. In California, 12% of students in K–12 schools are in special education (Jones, 2020); however, only 20% of these students are in special education classrooms on a comprehensive campus, and 3% of these students are in separate schools away from their school or district of residence (Petek, 2019). For this 3% of students who are most impacted and unable to be educated at their school of residence, research on distance-learning outcomes is extremely scarce. In fact, the researchers from the present study were unable to find any articles that specifically looked at distance learning in nonpublic schools (NPSs), let alone distance-learning studies in an NPS during an international emergency.

The COVID-19 pandemic and corresponding public health crisis initiated an unprecedented and rapid move to distance-learning platforms. Almost overnight, educators, students, and their families have found themselves in uncharted waters. The need for more research on distance learning and students with special needs, especially students with severe developmental disabilities and high behavioral needs, is quite apparent.

## Study 1

The purpose of Study 1 was to explore the social validity of distance-learning programs for students with severe developmental disabilities and high behavioral needs. Information was gathered from students’ families and their educators (teachers and paraeducators) on the feasibility and effectiveness of distance-learning programs. A survey with Likert-scale ratings, multiple-choice questions, and open-ended questions was utilized to measure parent and educator perceptions of the distance-learning program.

### Method

#### Educational Setting

##### The Brick-and-Mortar Setting

Prior to school closures, the students in this study attended a two-campus NPS program in Southern California certified by the California Department of Education (CDE). According to the CDE (2020), NPSs are specialized private schools that provide services to public school students with disabilities. The NPS in the present study primarily serves students with ASD, IDs, and other developmental, behavioral, and cognitive disabilities. One hundred twenty-six students, ages 5 through 22 years, are educated at the NPS. Each classroom consists of up to 12 students, a credentialed teacher, and anywhere from 3 to 12 paraeducators. Given the significant needs of the students, 82.5% of the students have one-to-one paraeducator support throughout the school day. In addition, the NPS utilizes a behavior-analytic framework for all educational services and employs 12 Board Certified Behavior Analysts to ensure that behavior analysis informs both school- and classroom-wide systems, as well as students’ individual behavior plans.

##### The Distance-Learning Setting

In mid-March, the rapidly evolving COVID-19 pandemic led the NPS’s administrative and teaching teams to quickly redesign their approach to teaching, services, and the way in which services and supports were delivered to the students. It was determined that a synchronous distance-learning model would be the best approach to serve the NPS’s student population. Within a 1-week time span, the distance-learning program was developed, finalized, disseminated through a training program, and launched to all staff. Training consisted of an initial 4-hr training with all teachers and support providers. Training included instruction on the online virtual platform used (www.zoom.us); the utilization of breakout rooms for one-to-one and small-group instruction; the technological systems needed, including how to share a screen; data collection procedures; curriculum and resource libraries available online; the implementation of IEP goals virtually; the completion of academic assessments remotely; and the development of classroom schedules and Zoom meeting codes for entrance into the classroom settings. Additionally, each week following the initial training, teachers and support providers would meet for 2 hr to troubleshoot and collaborate on resources that were most effective.

Zoom was selected as the online virtual platform. All Zoom sessions were kept private through meeting identification numbers and passwords, which were only provided to parents of currently enrolled students. All classroom assignments remained the same so that students were grouped with the same teacher and peers whom they were accustomed to on campus. The virtual school day was scheduled for all students from 9:00 a.m. to 3:00 p.m. Schedules varied by classroom and student but typically consisted of teacher-led group instruction in the morning and one-to-one instruction for all services related to their IEP in the afternoon. For group instruction, teachers would share their screens and students were able to participate by adding content to the shared lesson through the annotation feature or by utilizing the raise-hand feature and waiting to be called on. All one-to-one service meetings were held apart from the virtual group setting in virtual breakout rooms. During the one-to-one service meetings, the students worked with the service provider or paraeducator, and on occasion, a behavior team member. Academic and behavior goals, as well as all one-to-one speech and occupational therapy, were targeted during this time. The speech and occupational therapy teams also led group instruction times throughout the virtual school day to ensure that all students received collaborative service opportunities and all services as outlined in their IEPs.

#### Participants

The survey was sent to all parents and educators of students enrolled at the Southern California NPS offering the distance-learning program.

##### Parents

Inclusion criteria included three aspects: Participants (a) had to have a child who was enrolled in the NPS for 3 months or longer, (b) had to have a child who was currently participating in the NPS’s distance-learning program, and (c) had to have completed 90% of the survey items. Of the 182 parents whom the survey was sent to, 40 met these inclusion criteria and were included in the final analyses (21.9%).

##### Educators

To be included in the analyses, the educators must have (a) been employed at the NPS for a minimum of 3 months, (b) been providing instruction in the distance-learning program, and (c) completed 90% of the survey items. Of the 15 teachers whom the survey was sent to, 11 teachers met these inclusion criteria and were included in the final analyses (73.3%). Finally, of the 104 paraeducators whom the survey was sent to, 59 met these inclusion criteria and were included in the final analyses (56.7%).

##### Students Enrolled

Although the students enrolled were not surveyed, it is important to understand the developmental and behavioral needs of the students enrolled at the NPS. Students attending the NPS have no functional language or limited verbal abilities and low cognitive and adaptive profiles and demonstrate severe and dangerous challenging behavior. Such behaviors include severe physical aggression and self-injurious behavior that resulted in injury prior to enrollment, property destruction requiring replacement of technology or furniture or building repairs, and/or elopement into dangerous environments, including highways, private residences, and/or railroad crossings.

#### Instrumentation

The parent and educator (teacher and paraeducator) surveys were developed and hosted through SurveyMonkey (www.surveymonkey.com). Prior to completing the survey, participants were presented with an informed consent form. The parent survey consisted of four sections. The first section pertained to student demographics. The second section consisted of 11 statements related to the feasibility and effectiveness of the distance-learning program. Items 1–5 measured perceptions of distance-learning feasibility, and Items 6–11 measured perceptions of effectiveness. Participants rated their level of agreement with these statements using a 5-point Likert-type scale (1 = *strongly disagree*, 2 = *disagree*, 3 = *neutral*, 4 = *agree*, 5 = *strongly agree*). The third section of the survey consisted of two multiple-choice questions gauging the level of parent and educator support that their child required to participate in the distance-learning program. Lastly, the fourth section consisted of three open-ended questions where parents could share their experiences with the distance-learning program. The questions included what respondents liked and disliked about the distance-learning program, as well as the impact that it had on their child.

The educator survey mirrored the parent survey, consisting of the same four sections. However, instead of asking respondents to think about their child when completing the survey, respondents were asked to think about their students when completing the survey. Participants could choose not to answer any question, but they could not revisit questions after advancing in the survey.

#### Response Measurement and Data Analysis

The percentage of respondents who selected each response option was calculated per question for all multiple-choice items. The mean and standard deviation were calculated for all Likert-type survey items. Additionally, the percentage of agreement was calculated for each item by calculating the percentage of respondents who indicated agreement with the item (i.e., response of *agree* or *strongly agree*). The percentage agreement index is used as a supplemental analysis for Likert-scale satisfaction items and is another method for interpreting survey responses (Sauro, 2011). Acquiescence bias, or the tendency for people to agree rather than disagree, is prevalent with survey items (Kuru & Pasek, 2016). Therefore, items with a percentage agreement score of 75% or higher were interpreted as items with strong endorsement. This is a more conservative interpretation than a simple majority and has been used by other behavior-analytic researchers (e.g., Taylor et al., 2019).

Finally, open-ended questions were grouped based on common content and statements to determine anecdotal response themes. In order to be counted as a theme, at least two respondents had to have written content pertaining to that theme. Once the themes were developed, the first author coded each statement to determine which themes applied to each statement. The top three themes for each question were then determined for all three respondent types based on the number of statements that fell under each theme. The percentage of respondents who endorsed each theme was then calculated by dividing the number of respondents’ statements that endorsed each theme by the total number of respondents. Interobserver agreement checks were conducted for 100% of the comments. The second author independently coded all of the comments for each theme. If both coders coded the same statement under a theme, an agreement was counted. If one coder coded a statement under a theme, but the other coder did not code that statement under a theme, a disagreement was counted. Interobserver agreement was then calculated for each theme by dividing the number of agreements by the number of disagreements plus agreements and multiplying by 100. For parent responses to the question “What do you like about your child’s distance-learning program?” interobserver agreement was 100%. For parent responses to the question “What do you dislike about your child’s distance-learning program?” interobserver agreement averaged 78.0% (range 63.6%–100%). For parent responses to the question “How has the switch to distance learning affected your child?” interobserver agreement averaged 92.1% (range 75.0%–100%). For teacher responses to the question “What do you like about your students’ distance-learning program?” interobserver agreement averaged 73.3% (range 66.7%–80%). For teacher responses to the question “What do you dislike about your students’ distance-learning program?” interobserver agreement averaged 87.5% (range 75.0%–100%). For teacher responses to the question “How has the switch to distance learning affected your students?” interobserver agreement averaged 94.4% (range 83.3%–100%). For paraeducator responses to the question “What do you like about your students’ distance-learning program?” interobserver agreement averaged 92.0% (range 80.0%–100%). For paraeducator responses to the question “What do you dislike about your students’ distance-learning program?” interobserver agreement averaged 88.2% (range 77.8%–100%). For paraeducator responses to the question “How has the switch to distance learning affected your students?” interobserver agreement averaged 88.8% (range 80.0%–88.9%).

#### Procedure

Parents were sent a letter informing them of the current study via Remind, an electronic-based communication system for schools. The parent letter included the survey link. Educators were emailed information about the survey and the survey link via their work emails. Upon opening the link, the online consent form was presented.

### Results

Tables 1, 2, 3, and 4 depict the results from the parent survey. Table 1 displays the demographic characteristics of the parents’ children. Parents reported that 25.0% (*n* = 10) of their children were in elementary classrooms, 35.0% (*n* = 14) of their children were in middle school classrooms, 25.0% (*n* = 10) of their children were in high school classrooms, and 15.0% (*n* = 6) of their children were in adult transition classrooms. The majority of the parents reported that their child was enrolled in the NPS for more than 3 years (47.5%, *n* = 19), and 42.5% (*n* = 17) reported that their child was enrolled for more than 1 year but less than 3 years. Only 10% (*n* = 4) reported that their child had been enrolled for less than 1 year. The majority of the parents (82.5%, *n* = 33) also reported that their child required one-to-one support per their IEP.

**Table 1:** Parent-Reported Child Demographics

Item	*n*	%
Educational level		
Elementary	10	25.0
Middle school	14	35.0
High school	10	25.0
Adult transition	6	15.0
Enrollment duration		
Less than 3 months	0	0.0
Since the start of the school year	4	10.0
For more than 1 year, but less than 3 years	17	42.5
For 3 years or more	19	47.5
Student requires 1:1 support per their IEP		
Yes	33	82.5
No	7	17.5
Attending NPS’s distance-learning program at time of study		
Yes	40	100.0
No	0	0.0

*Note.* IEP = Individualized Education Plan; NPS = nonpublic school.

**Table 2:** Frequency and Percentage of Parents Indicating Level of Support That Student Needs During Distance-Learning Program

Level of support needed	*n*	%
Level of parent assistance needed		
None. My child is independent.	0	0.0
Minimal. Once I get them going, my child is fine.	3	7.5
Occasional. They need me to check in regularly.	1	2.5
A lot. I have to sit right next to them.	36	90.0
Level of staff assistance needed		
None. My child is independent.	0	0.0
Minimal. Staff needs to check in now and then.	5	12.5
Occasional. Staff needs to check in regularly.	11	27.5
A lot. My child is not successful unless they are with staff in a virtual breakout room.	24	60.0

**Table 3:** Parents’ Level of Agreement With Statements Regarding Their Child’s Distance-Learning Program

Statement	*M* (*SD*)	% agree
I have enough time to implement my child’s distance-learning program.	3.15 (1.11)	37.5
The Zoom platform is easy to operate when implementing my child’s distance-learning program.	3.83 (1.07)	75.0
I have all the tools necessary to support my child during their distance-learning program.	3.43 (1.02)	47.5
My occupation or other responsibilities make it difficult for me to assist my child in participating in their distance-learning program.	3.43 (1.24)	57.5
My child’s behaviors impede their ability to engage in their distance-learning program.	3.38 (1.37)	50.0
My child is engaged during their distance-learning program.	3.25 (1.09)	37.5
My child is learning during their distance-learning program.	3.48 (0.97)	47.5
My child is obtaining educational benefits by participating in their distance-learning program.	3.43 (1.20)	47.5
My child continues to make progress on their IEP goals through their distance-learning program.	3.28 (1.14)	42.5
My child spends enough time in their distance-learning program to meet their learning needs.	3.00 (1.32)	32.5
The switch from in-person interactions to virtual interactions between my child and the classroom teacher has negatively impacted my child’s education.	3.45 (1.24)	55.0

*Note.* IEP = Individualized Education Plan.

**Table 4:** Top Three Themes Reported by Parent Respondents in Open-Ended Survey Questions

Theme	Example	% (frequency)
What do you like about your child’s distance-learning program?		
Continued socialization	“Having the other students visible and so many familiar staff involved (both trading off and just visible) has really made a big difference in keeping our child connected and enthusiastic.”	20.0 (8)
Provides structure	“Keep’s him busy, instead of playing video games.”	17.5 (7)
Program visibility	“I am able to see how the class is conducted. Meeting and putting names to faces of the staff, witnessing how everyone interacts and what the strengths are, able to witness how great his teachers and aide are. I feel that I am more aware of my son’s abilities and he is so motivated by my presence helping him but best of all I know more about his education and the staff now.”	17.5 (7)
What do you dislike about your child’s distance-learning program?		
Requires high level of parent support	“That I have to be next to him to make sure he doesn’t elope and makes sure he listens. Lots of prompting going on. I have work and have 2 other kids homeschooling that has to wait until our [distance learning] is done. It’s hard.”	25.0 (10)
Child not engaged	“It is really hard for my son to sit and attend to what is going on. He wants to see everyone and visit, but not sit and attend to the lesson.”	22.5 (9)
Limited social interactions	“He doesn’t get to interact in person with his peers or staff. He looks forward to this and I feel it is a very important part of his day and his learning. He misses his school program and doesn’t understand why he can’t go. He asks daily to go to school. School is the only place he gets to be around his peers. I feel like this is having an impact on his mental and physical well-being. I know I miss having my friends around and can’t imagine how he feels.”	17.5 (7)
How has the switch to distance learning affected your child?		
Increase in challenging behaviors	“He had a very difficult time for the first several weeks. He began to engage in maladaptive behaviors that he hadn’t had in many months. It was very challenging, affecting the whole family.”	17.5 (7)
Missed friends/staff	“He misses going to school and be with his friends and staff.”	17.5 (7)
Negative impact on progress	“He has lost skills, despite everyone’s best efforts.”	10.0 (4)

Table 2 indicates the level of support that their child needed during the distance-learning program. The majority of parents reported that their child required that the parent sit right next to them in order to attend the distance-learning program (90.0%, *n* = 36). Fewer reported that their child needed the parent to check in regularly (2.5%, *n* = 1) or simply get them started (7.5%, *n* = 3). Additionally, the majority of parents reported that their child required a staff member to be in a breakout room with their child in order for their child to attend the distance-learning program (60.0%, *n* = 24). Fewer reported that their child simply needed staff to regularly check in (27.5%, *n* = 11) or staff to check in now and then (12.5%, *n* = 5).

Table 3 depicts the summary of results from the parent survey regarding the feasibility and effectiveness of their child’s distance-learning program. Of the 12 items, only 1 has a mean score of 4 or above or an agreement score of 75% or above. Parents agreed that the Zoom platform was easy to operate when implementing their child’s distance-learning program (*M* = 3.83, 75.0% agreement). All of the other items had a mean score below 4, indicating that on average, respondents did not agree with these statements. In general, parents rated both the feasibility (e.g., having the tools necessary to support their child, balancing their child’s distance-learning program with their other responsibilities) and effectiveness (e.g., child’s level of engagement, child’s progress on their IEP goals) of the distance-learning program as neutral.

Parents also answered open-ended questions regarding their child’s distance-learning program (see Table 4). In response to the question “What do you like about your child’s distance-learning program?” the top three themes were continued socialization (20.0%, *n* = 8), the structure that the program provided (17.5%, *n* = 7), and the parents’ increased visibility into their child’s education (17.5%, *n* = 7). In response to the question “What do you dislike about your child’s learning program?” the top three themes were that the parents had to provide a high level of support (25.0%, *n* = 10), that their child was not engaged (22.5%, *n* = 9), and that there were limited social interactions (17.5%, *n* = 7). In response to the question “How has the switch to learning affected your child?” the top three themes were that there was an increase in challenging behaviors (17.5%, *n* = 7), that their child missed their friends and staff (17.5%, *n* = 7), and that there was a negative impact on their child’s progress (10.04%, *n* = 4).

Tables 5, 6, 7, and 8 depict the results from the teacher survey. Table 5 displays the demographic characteristics of the teachers. Of these 11 respondents, 27.3% (*n* = 3) taught at the elementary level, 36.4% (*n* = 4) taught at the middle school level, 27.3% (*n* = 3) taught at the high school level, and 9.1% (*n* = 1) taught at the adult transition level.

**Table 5:** Teachers’ and Paraeducators’ Reported Grade Level Taught

Grade level	Teachers		Paraeducators	
	*n*	%	*n*	%
Elementary	3	27.3	12	20.3
Middle school	4	36.4	20	33.9
High school	3	27.3	12	20.3
Adult transition	1	9.1	15	25.4

**Table 6:** Frequency and Percentage of Teachers and Paraeducators Indicating Level of Support That Student Needs During Distance-Learning Program

Level of support needed	Teachers		Paraeducators	
	*n*	%	*n*	%
Level of parent assistance needed				
None. My student is independent.	0	0.0	2	3.4
Minimal. Once their parents get them going, my student is fine.	0	0.0	6	10.2
Occasional. My student needs their parent to check in regularly.	5	45.5	11	18.6
A lot. My student needs their parent to sit right next to them.	6	54.6	40	67.8
Level of staff assistance needed				
None. My student is independent.	0	0.0	4	6.8
Minimal. Staff needs to check in now and then.	0	0.0	8	13.6
Occasional. Staff needs to check in regularly.	6	54.6	14	23.7
A lot. My student is not successful unless they are with staff in a virtual breakout room.	5	45.5	33	55.9

**Table 7:** Teachers’ and Paraeducators’ Level of Agreement With Statements Regarding Their Students’ Distance-Learning Program

Statement	Teachers		Paraeducators	
	*M* (*SD*)	% agree	*M* (*SD*)	% agree
I have enough time to teach each of my students’ IEP goals during their distance-learning programs.	3.91 (0.79)	63.6	3.81 (0.98)	69.5
The Zoom platform is easy to operate when implementing my students’ distance-learning program.	4.00 (0.60)	81.8	3.80 (0.93)	67.8
I have all the tools necessary to support my students during their distance-learning program.	3.82 (1.03)	72.7	3.37 (0.97)	45.8
My home life or other responsibilities make it difficult for me to lead my students’ distance-learning program.	2.73 (1.48)	36.4	2.49 (1.32)	22.0
My students’ behaviors impede their ability to engage in their distance-learning program.	3.27 (1.05)	36.4	2.85 (1.16)	35.6
My students are engaged during their distance-learning program.	3.82 (0.83)	72.7	3.58 (0.96)	57.6
My students are learning during their distance-learning program.	4.00 (0.85)	81.8	3.83 (0.89)	69.5
My students are obtaining educational benefits by participating in their distance-learning program.	4.36 (0.88)	90.9	4.00 (0.86)	79.7
My students continue to make progress on their IEP goals through their distance-learning program.	3.82 (0.83)	72.7	3.64 (0.99)	61.0
My students spend enough time in their distance-learning program to meet their learning needs.	3.18 (1.03)	36.4	3.17 (1.15)	44.1
The switch from in-person interactions to virtual interactions between my students has negatively impacted my students’ education.	3.27 (1.14)	54.5	2.75 (1.10)	18.6

*Note.* IEP = Individualized Education Plan.

**Table 8:** Top Tree Themes Reported by Teacher Respondents in Open-Ended Survey Questions

Theme	Example	% (frequency)
What do you like about your students’ distance-learning program?		
Increase staff collaborations	“The collaboration between teachers allows for different teaching styles, which allow the students to learn in different ways.”	36.4 (4)
Continued social contact	“I like that my students are still able to see their teachers and classmates.”	27.3 (3)
What do you dislike about your students’ distance-learning program?		
Decrease social interactions	“Personal interaction and engagement with students are not as good as typical school environment.”	36.4 (4)
Students not attending distance learning	“The biggest disadvantage would be that a lot of the students haven’t logged in to participate. Parents are either working from home, don’t have access to the internet, or are concerned with behaviors that may occur if their child participates.”	36.4 (4)
Difficulty training parents/home staff	“Sometimes it is hard to teach or train the students’ home staff or caregivers about the expectations on each goal (prompting).”	18.2 (2)
How has the switch to distance learning affected your students?		
Some benefits	“Some students have really thrived. They are relaxed, present, and engaged throughout the lesson.”	45.5 (5)
Students absent	“I feel like there will be a big impact on the students who have not been able to access distance learning when we return to campus.”	36.4 (4)
Had to learn new skills	“My students have had to learn some new skills, and overall they are doing very well.”	18.2 (2)

Table 6 depicts the level of support the teachers’ students needed during their distance-learning program. All of their students required either regular check-ins from the parent (45.5%, *n* = 5) or a parent sitting right next to the student (54.6%, *n* = 6). In addition, the teachers reported that their students required the staff to check in regularly (54.6%, *n* = 6) or the staff to be in a breakout room with their students (45.5%, *n* = 5).

Table 7 depicts the summary of results from the teacher surveys regarding the feasibility and effectiveness of their students’ distance-learning program. Of the 12 items, 3 were endorsed by the teachers (i.e., a mean score of 4 or above or an agreement score of 75% or above). Teachers agreed that the Zoom platform was easy to operate when implementing their students’ distance-learning program (*M* = 4.0, 81.8% agreement), that their students were learning during their distance-learning program (*M* = 4.0, 81.8% agreement), and that their students were obtaining educational benefits by participating in their distance-learning program (*M* = 4.36, 90.9% agreement). All of the other items had a mean score below 4, indicating that, on average, respondents did not agree with these statements and responded neutrally toward them. Of interest, the teachers’ lowest score (*M* = 2.73, 36.4% agreement) was for the item “My home life or other responsibilities make it difficult for me to lead my students’ distance-learning program,” reflecting that more respondents disagreed with this item (*n* = 6) than agreed with the item (*n* = 4).

Teachers also answered open-ended questions regarding their students’ distance-learning program (see Table 8). In response to the question “What do you like about your student’s distance-learning program?” the top themes were increased staff collaboration (36.4%, *n* = 4) and continued social contact (27.3%, *n* = 3). In response to the question “What do you dislike about your student’s distance-learning program?” the top three themes were that there were decreased social interactions (36.4%, *n* = 4), that a lot of students were not attending distance learning (36.4%, *n* = 4), and that it was difficult to train parents and home staff (18.2%, *n* = 2). In response to the question “How has the switch to distance learning affected your students?” the top three themes were that there were some benefits from distance learning (45.5%, *n* = 5), that there was a negative impact on the students who were absent from distance learning (36.4%, *n* = 4), and that students had to learn new skills to participate in distance learning (18.2%, *n* = 2).

Tables 5, 6, 7, and 9 depict the results from the paraeducator survey. Table 5 displays the demographic characteristics of the paraeducators. Of these 59 paraeducators, 20.3% (*n* = 12) were in elementary classrooms, 33.9% (*n* = 20) were in middle school classrooms, 20.3% (*n* = 12) were in high school classrooms, and 25.4% (*n* = 15) were in the adult transition program.

**Table 9:** Top Three Themes Reported by Paraeducators in Open-Ended Survey Questions

Theme	Example	% (frequency)
What do you like about your students’ distance-learning program?		
Parent involvement	“I also appreciate the parents’ willingness to learn. This time has allowed parents to learn more about their child and how they can best support them at home. This has created a greater bond between student–parent relationships.”	15.3 (9)
Access to virtual tools	“Some materials are better presented on the computer and other learning programs.”	15.3 (9)
Fewer distractions	“I like how easy it is to have a private room with my student without the distraction of other students/staff.”	10.2 (6)
What do you dislike about your students’distance-learning program?		
Parents interfering	“Sometimes parents overprompt and I worry about my student becoming prompt dependent.”	13.6 (8)
Difficult to address challenging behaviors	“I dislike my students’ ability to elope or be off task without my presence there to keep them on task. I also dislike not being aware of their entire environment and not knowing if there was a trigger for a behavior off of the screen.”	11.9 (7)
Need to be face to face for some goals	“The distance learning does not allow the students the full 1:1 support that they require. It appears that distance learning is ineffective when teaching new skills or running IEP goals, as the students with high behavioral needs and severe learning disabilities seem to not pay attention or engage with a computer screen regardless of whether the staff is on screen or not. It appears as though they do not fully understand the concept of online learning, as most require hand-over-hand assistance or physical prompting when on campus, in person.”	10.2 (6)
How has the switch to distance learning affected your students?		
Made things worse	“My student has regressed behaviorally and academically.”	18.6 (11)
Seen improvements	“It’s been better for my student. Seems to be more engaged and less challenging behaviors.”	15.3 (9)
No impact	“None. My students come to virtual school almost every day, and we target most of the goals on a daily basis.”	13.6 (8)

*Note.* IEP = Individualized Education Plan.

Table 6 depicts the level of support their students needed during their distance-learning program. The majority of respondents reported that their students required a parent to sit right next to the student (67.8%, *n* = 40). Fewer paraeducators reported that their student needed regular parent check-ins (18.6%, *n* = 11). Even fewer paraeducators reported that their students simply needed parent assistance to get their student going (10.2%, *n* = 6) or no assistance at all (3.4%, *n* = 2) from a parent. In addition, the level of staff assistance required was assessed. The majority of paraeducators reported that their student needed to be in a breakout room with staff (55.9%, *n* = 33), whereas 23.7% (*n* = 14) reported that their student required regular check-ins, 13.6% (*n* = 8) simply required a staff member to get them going, and 6.8% (*n* = 4) did not require staff assistance.

Table 7 depicts the summary of results from the paraeducator surveys regarding the feasibility and effectiveness of their students’ distance-learning program. Of the 12 items, only 1 has a mean score of 4 or above or an agreement score of 75% or above. Paraeducators agreed that their students were obtaining educational benefits by participating in their distance-learning programs (*M* = 4.0, 79.7% agreement). All of the other items had a mean score below 4, indicating that, on average, respondents did not agree with these statements. Of particular interest, the paraeducators scored the three negatively worded items the lowest, indicating more positive attitudes toward distance learning (i.e., they disagreed that it was difficult for them to balance their home life with leading their students’ distance-learning program, that their students’ behaviors impeded the students’ ability to engage in distance learning, and that the switch from in-person interactions to virtual interactions with their students negatively impacted their students’ education, all items with means below 2.85 and below 35.6% agreement).

Lastly, paraeducators also answered open-ended questions regarding their students’ distance-learning program (see Table 9). In response to the question “What do you like about your student’s distance-learning program?” the top three themes were parent involvement (15.3%, *n* = 9), access to virtual tools (15.3%, *n* = 9), and fewer distractions with distance learning (10.2%, *n* = 6). In response to the question “What do you dislike about your student’s distance-learning program?” the top three themes were parents interfering (13.6%, *n* = 8), the difficulty in addressing the students’ challenging behaviors (11.9%, *n* = 7), and the need to be face to face for some of the goals (10.2%, *n* = 6). In response to the question “How has the switch to distance learning affected your students?” the top three themes were conflicting. The most widely endorsed theme was that distance learning had made things worse (18.6%, *n* = 11). However, the next two highest themes were that they had seen improvements (15.3%, *n* = 9) and that distance learning had no impact on the students (13.6%, *n* = 8).

### Discussion

Study 1 examined the social validity of distance-learning programs for students with severe developmental disabilities and high behavioral needs. Parent, teacher, and paraeducator surveys were analyzed to determine perceptions of feasibility and effectiveness for students ages 5–22 years who attended an NPS in Southern California.

An analysis of feasibility ratings revealed that, overall, parents agreed that although aspects of the distance-learning program were feasible (i.e., the Zoom platform was easy to operate), it did impact typical routines and demanded adjustments. Overall, parent ratings on the feasibility survey items indicated that parents were neutral toward the feasibility of their children’s distance-learning program. The scores from the parents who felt like the program was feasible were offset by the scores from the parents who felt like the program was not feasible, creating overall means in the neutral range. Further research should look at the differences between these two groups to determine what variables make it likely for some parents to respond favorably toward distance learning and others to respond unfavorably. An analysis of the open-ended responses indicated that an area requiring further attention is the high level of parent involvement required. The majority of parents, teachers, and paraeducators all indicated that the students needed a lot of parent assistance (e.g., most parents had to sit right next to their children throughout the day). It is quite possible that families who could accommodate the high level of parental assistance rated the feasibility as more favorable than those families who could not easily accommodate such a high level of parental involvement (e.g., families in which both parents had a full-time job or families with multiple children).

Overall, teachers and paraeducators were also neutral toward most aspects of the feasibility of the distance-learning program. Of note, however, is that both teachers and paraeducators did not report that it was difficult to balance their home life with leading their students’ distance-learning programs. This is of particular interest because the educators in this study had to be on the distance-learning platform for most of the regularly scheduled school day. Future research should continue to investigate this, especially given conversations surrounding the difficulty that parents face nationwide with balancing their job responsibilities while facilitating their own children’s distance learning (Adams & Todd, 2020).

Parents’ ratings of the effectiveness of distance learning were also neutral; however, their responses to the open-ended questions indicated several general themes. First, parents indicated that a benefit of distance learning was continued socialization (i.e., their children still had contact with their teachers and got to see their classmates). However, parents also indicated that distance learning limited their child’s social interactions. Together, it seems like parents appreciated that their children got to interact with their classmates during distance learning, but recognized that these interactions were inferior when compared to the in-person interactions that their children received prior to distance learning. Given the importance of social skills and the potential impacts on other areas of development, including academic achievement, social acceptance, and comorbid diagnoses (Bellini, 2006; La Greca & Lopez, 1998; Tantam, 2000; Welsh et al., 2001), this is an important consideration when looking at distance-learning programs and their impact on this population.

Similar to parents, teachers’ and paraeducators’ overall survey scores were generally neutral when rating the effectiveness of their students’ distance-learning program. One important difference, however, is that both teachers and paraeducators agreed that their students were obtaining educational benefits by participating in their distance-learning program. Parents, on the other hand, were neutral toward this item. This is an important distinction. Whereas the majority of educators recognized the benefits of distance learning for their students, the majority of parents did not. A limitation of this study is that disparate sample sizes preclude statistical analyses comparing the results of the three groups to determine whether these differences were significant. This is an opportunity for future research to investigate.

The educators’ open-ended responses highlight certain nuances regarding the effectiveness of distance learning. Responses suggest several unique benefits to distance learning because it was conducted in the students’ homes, such as increased opportunities for parent involvement. Paraeducators also noted that several of the students’ home settings presented fewer distractions than classrooms full of other students and adults. On Zoom, paraeducators could work with a student one to one in a breakout room, whereas in a classroom, it was more difficult to find a location away from the other students. Another unique benefit observed by the educators was related to the technological aspects of distance learning. Teachers noted that distance learning forced the students to learn new skills (e.g., how to independently attend a Zoom meeting), and paraeducators described how it was easier to target certain goals on the computer than it had previously been in the brick-and-mortar setting. These comments underscore some of the technological benefits of distance learning and contribute to current conversations on how the transition to distance learning will have an everlasting impact on how education is delivered, even when brick-and-mortar schools reopen (Li & Lalani, 2020). Of note, however, is that the unique characteristics of distance learning that lead to these advantages also come with distinct disadvantages. Teachers described the difficulties training the students’ parents over Zoom, and paraeducators commented that parents would often overprompt their children. Further, distance learning made it difficult to address the children’s challenging behaviors. Even though distance learning and the access to virtual tools made it easier to address certain goals, other goals could only be addressed face to face. Overall, the results highlight the nuanced reactions to and perceptions of distance learning. Educators recognized that distance learning encompassed both unique benefits and disadvantages, especially when working with a population of students with several developmental disabilities and high behavioral needs.

Again, given the COVID-19 pandemic and school closures that caused almost all students across most of the United States to transition to distance-learning programs, the reported impact on this population is concerning. Although there is research to support that for some students in mainstream settings, distance learning may be equally beneficial (Barbour & Mulcahy, 2009) or perhaps superior to in-person instruction (Hart et al., 2019), less is known about students with severe developmental disabilities and high behavioral needs. The present study begins to provide information on the impacts of distance learning on this population, suggesting that parents’ and educators’ attitudes toward the feasibility and effectiveness of distance-learning programs are generally neutral. Whereas educators felt like their students were obtaining some educational benefits from participating in distance learning, parents did not share this belief.

Despite the informative findings from the survey data, limitations of the present study exist. One limitation of this study is that parents whose children were unable to participate in the distance-learning program were not included in the study. Future research should investigate how many children did not participate in distance learning during the COVID-19 school closures, and what barriers prevented these children from participating. It may be that participating at any level was not feasible for these families, therefore indicating that in-person instruction is needed. The exclusion of those who did not participate skews the survey results, as those who were unable to participate may have provided lower ratings on all effectiveness and feasibility items. Alternatively, perhaps parents whose children did not participate had lower levels of stress, observed decreased rates of challenging behaviors given the lack of educational demands, and had more flexible schedules to tend to other responsibilities.

An additional limitation of Study 1 is that parents, teachers, and paraeducators from only one NPS were included in the present study. Although the NPS had two campuses in two different counties in California, the study is not representative of this population as a whole. Only 20% of California students are in special education classrooms, and even fewer (3%) are placed in separate schools such as the NPS examined in the study. Therefore, generalization of findings to the special education community, and their distance-learning programs as a whole, cannot be made.

Finally, when interpreting the results from Study 1, it should be noted that data collection occurred during a national pandemic. Therefore, there exists a possible threat to internal validity—namely, history. It is difficult to determine whether participants were responding to distance learning solely or responding to distance learning during a pandemic. It is possible, and quite likely, that participants had additional stressors during this time that influenced their responses. Similarly, the immediacy of the transition to distance learning and the lack of a planning period during the pandemic potentially influenced participants’ responses. Therefore, more work is needed to examine similar distance-learning programs during a time that is more representative of “normal life.”

Despite the limitations of the study, the data add to a small body of literature investigating the feasibility and effectiveness of distance-learning platforms for students with severe developmental delays and high behavioral needs. Such studies are imperative, as there is not a lot of information regarding distance learning and this population. Further, during a pandemic such as COVID-19, where distance learning becomes the only option, it is essential to understand the impacts that distance learning may have on families, educators, and students representing this unique population.

## Study 2

The purpose of Study 2 was to investigate the impact that transitioning to a distance-learning program had on the academic progress of students with severe developmental disabilities and high behavioral needs. In particular, the data from students’ IEP goals pre- and posttransition to the distance-learning program were analyzed.

### Method

#### Participants

To determine the impact that the transition to a distance-learning program had on students with severe developmental delays and high behavioral needs, data on students’ IEP goals were examined. All academic and behavior replacement goals for 42 students enrolled in the distance-learning program for the NPS were included in the analysis. Students’ data were included in the analysis if the students were participating in the distance-learning program a minimum of 75% of the time it was offered. Eighty-four students were excluded from Study 2 because they did not meet this attendance requirement. Additionally, the goals must have been implemented for a minimum of 3 weeks prior to the transition to distance learning and also implemented for a minimum of 3 weeks after the transition to distance learning. If goals were modified, changed, or discontinued as a result of an annual or triennial IEP meeting, the student’s data were excluded. Only goals that utilized percentage opportunity data collection procedures were included in the analyses. The following statement is an example of the type of behavior goals included in the analysis:

The student will follow two-step directives relating to navigating his environment and/or task engagement (e.g., “grab a pencil and go your to desk”) as evidenced by initial directive engagement within 10 s and completion in the absence of challenging behavior on 85% of opportunities, over 2 consecutive weeks, across three settings, two individuals, and a minimum of 10 two-step directives as measured by data collection.

The following statement is an example of the type of academic goals included in the analysis:

When given a picture of an object with a designated price, the student will round up to the next dollar amount and select the correct amount from a field of three, with 80% accuracy, across 3 consecutive school days and three settings, as measured by data collection.

A total of 419 goals were analyzed based on these inclusion criteria.

#### Procedure

Students’ performance on their IEP goals was collected via an electronic data collection software called Catalyst (www.datafinch.com). This program is secure, compliant with the Health Insurance Portability and Accountability Act, and was already part of the students’ traditional educational program. Data were collected as part of standard clinical practices in the brick-and-mortar program, as well as during the distance-learning program. Data collection procedures did not change with the exception of the interactions being virtual rather than in person.

### Results

A paired-samples *t* test was conducted to determine if the students’ mean performance on their IEP goals during distance learning differed from their mean performance on their IEP goals during in-person instruction. Data for 42 students were included in the analysis. The number of IEP goals included per student ranged from 1 to 19 (*M* = 9.98, *SD* = 4.88). The average score on the students’ IEP goals during distance learning (*M* = 73.94, *SD* = 1.38) was almost the same as the average score on the students’ IEP goals during in-person instruction (*M* = 74.98, *SD* = 1.28). This difference was not statistically significant, *t*(419) = −.97, *p* > .05.

Further analyses were conducted to determine if the null results obtained from the *t* test were because the students’ performance on their goals did not change from in-person learning to distance learning, or because the goals that showed improvement were offset by the goals that did not show improvement, thus canceling each other out. Each goal was categorized as “no change,” “regression,” or “progress” by subtracting the student’s mean performance on the goal during distance learning from their mean performance on the goal during in-person learning to obtain a difference score. If the student’s mean performance dropped by more than 10 percentage points (difference score less than −10), then the goal was categorized as “regression.” If the student’s mean performance improved by more than 10 percentage points (difference score greater than 10), then the goal was categorized as “progress.” Goals that changed by 10 or fewer percentage points were categorized as “no change.” See Table 10 for the number and percentage of goals in each category. Approximately half of the goals were categorized as “no change” (47.0%). Of the remaining goals, approximately a quarter were categorized as both “progress” (27.0%) and “regression” (26.0%).

**Table 10:** Number and Percentage of Individualized Education Plan Goals by Category

Category	*n*	%
No change	197	47.0
Regression	109	26.0
Progress	113	27.0

Last, a chi-square analysis was conducted to determine whether the distribution of goals in each category was expected. Results indicate that the proportion of goals in each category was not as expected by chance, χ^2^(2, *N* = 419) = 35.36, *p* < .01. More goals were categorized as “no change” than “progress” or “regression,” and this difference was statistically significant.

### Discussion

Study 2 investigated the impact that transitioning to a distance-learning program had on the progress on IEP goals of students with severe developmental disabilities and high behavioral needs. To expand on the effectiveness ratings from Study 1, the effectiveness of the distance-learning program was further analyzed by utilizing a paired-samples *t* test to compare students’ mean performance on their IEP goals with in-person instruction to their performance during the distance-learning program. Although there was a slight decrease in the overall mean, the difference was not statistically significant. Further analyses showed that the students’ performance did not change from in-person learning to distance learning for almost half of the goals, suggesting these skills maintained. Of the remaining goals, about a quarter showed reductions in performance, whereas the last quarter showed improvement. It is important to note that this suggests that the majority of goals measured showed maintenance or improvement. This is of important consideration, given the high level of supports, services, and curricular modifications per IDEA that this population requires in order to access their education (Lipkin & Okamoto, 2015). It is promising that a population that typically thrives on structure and routine maintained or continued to make progress on a majority of academic and behavior goals during a major disruption to their educational program. It is hypothesized that the quantity of time in their distance-learning program paired with programming familiar stimuli in the virtual classroom (e.g., teacher, peers, curriculum, schedule) facilitated maintenance on their IEP goals. Although many might find these results promising, others would justifiably question whether we should be satisfied with “maintenance.” In fact, the Supreme Court spoke directly to this concern in the ruling for *Endrew F. v. Douglas County School District* (2017). According to the ruling, students must be making expected progress toward annual IEP goals. There is an important distinction between progress and maintenance, and although it is promising that students only regressed on 25% of their goals during the transition to distance learning, maintenance is not the end goal. Although more research is needed to fully understand the effects that distance learning has on a severely impacted population, these findings are an informative first step.

Despite the promising findings demonstrated through performance on IEP goals, limitations of Study 2 are important to consider. One major limitation of Study 2 is that we do not have IEP goal performance data for students who did not participate in the distance-learning program or for students who participated for less than 75% of the time. Although students who did participate maintained or made progress on the majority of their IEP goals during distance learning, we cannot say that this is due to the distance-learning program, given we do not know the performance levels of those who did not participate. It is possible that those students would demonstrate similar performance on their goals in the absence of any program delivery. This shortcoming therefore limits the conclusions we are able to draw from Study 2.

A further limitation of Study 2 is that there are no interobserver agreement data for the students’ data during distance learning. Although it is standard practice at the NPS to take interobserver agreement data regularly, the limitations of the distance-learning program and the timeline in which it was developed and executed prevented consistent interobserver agreement data collection. Without this data, it is difficult to conclude that all goals that were included represent accurate data.

## General Discussion

Findings from Studies 1 and 2 contribute to a much-needed literature base, suggesting that although parents remain mostly neutral in their opinions regarding the feasibility and effectiveness of distance learning, teachers and paraeducators did feel that their students were receiving educational benefits from participating. In fact, even though neutral findings are generally not given much attention, in this case the parents’ neutral attitudes toward distance learning warrant further investigation. Given the shift in daily routines and responsibilities that came with an unexpected and sudden transition to distance learning, the fact that parent ratings were not more negative is interesting and promising for distance-learning programs in general. Further analyses of individual academic and behavior goals also indicate that students maintained skills for half of their goals and made progress on an additional quarter of their goals. This is noteworthy, especially given that this population often demonstrates regression in skills with interruptions to treatment (Allinder & Eicher, 1994; Tilley et al., 1986).

Caution should be taken before generalizing the findings of this study to other distance-learning programs. The distance-learning program under investigation in the current study strove to emulate the number of instructional minutes that students received in the brick-and-mortar setting. Just like when school was in person, students had access to their teacher for 6 hr per day. If a student received one-to-one support in person, they also received one-to-one support in their distance-learning program. Although information on other distance-learning programs after COVID-19 is sparse, reports suggest that many programs have certainly fallen short of this practice (Barnum & Bryan, 2020). Many students across the nation saw their teacher for 1 hr a week, and others not at all. Even less is known about special education programs in the era of distance learning during the COVID-19 pandemic. Therefore, caution must be taken before generalizing these results to other NPSs and even more so when generalizing to distance learning for special education students enrolled in public schools. Furthermore, it is difficult to determine if responses on the survey solely reflected distance learning or distance learning during an international emergency. The additional stressors on families may have altered their perception of distance learning.

Although students who attended the distance-learning program in the current study maintained or made progress on the majority of the goals included, it is not clear what would have happened to them had their educational minutes been significantly cut. Research on school breaks and students with special needs shows that regression does occur and duration to recoup skills lost varies by duration of break and severity of disability (Allinder & Eicher, 1994; Tilley et al., 1986). This implies that the impact of significantly cutting access to education for extended periods of time for students with severe developmental disabilities and high behavioral needs, as we have seen with the COVID-19 pandemic, will have dire consequences for these students and their families.

The present study found that distance learning forced the school and home settings to collide, and although this came with important benefits, it also resulted in some difficulties. Parents, teachers, and paraprofessionals all spoke to the fact that the distance-learning program resulted in increased parent visibility into their child’s educational program. Parents were able to see what their child was able to do academically and how to manage challenging behaviors more effectively. This created consistency of care and increased collaboration across the home and school environments. An additional benefit was that teachers and paraeducators had an accessible means to provide parent training. During traditional brick-and-mortar school sessions, parents often reported difficulties coming in for parent-training opportunities. Distance learning allowed for an alternative means to access parent training, and efforts should be made to continue access to parent training even when schools reopen their school sites. Although these benefits were extremely helpful, paraeducators and teachers also reported that parents’ involvement became problematic at times. For example, through no fault of their own, parents tended to overprompt and not follow prompt hierarchies outlined in the students’ Behavior Intervention Plan and learning plans. They often provided answers to their student prior to allowing their child to answer independently. This is understandable given the intensity of training that the educators have received in the strategies necessary to educate these students as compared to the minimal training that parents have received. Additionally, although parents reported that they enjoyed the participation, they also reported that it was challenging to find a work–home life balance and that the current setup was not sustainable.

Future research examining the feasibility and effectiveness of distance-learning delivery models for individuals with severe developmental disabilities and high behavioral needs is needed to fully understand the impact on this population. One area that warrants investigation is the specific impact that distance learning has on the rates and topographies of challenging behaviors. The present study only reviewed skill acquisition data, as observation of challenging behavior on the Zoom platform was difficult and unreliable. It is important to understand whether challenging behaviors increase, decrease, or remain stable during disruptions to typical educational practices and distance-learning mediums. Similarly, future research should include an experimental analysis that allows for direct comparison of in-person instruction with distance learning for students with severe disabilities and high behavioral needs. Without a direct comparison, we will not fully understand the effects that transitioning to distance learning has on this population.

In addition to efforts to more fully understand the feasibility and effectiveness of distance learning, more work is needed to understand best practices and to develop evidence-based protocols for distance-learning programs. Recently, California passed Senate Bill 98, which requires local education agencies to include a description of emergency procedures should distance learning be required in all student IEP documentation. Although advanced planning is certainly beneficial, we must understand what is needed to ensure student success with such programs. It will be important for future research to examine what the best practices for distance learning are to minimize regression and to ensure students’ success with this education modality.

Finally, as with the research on distance learning with the general education population, research is needed on any potential mediators and moderators that impact student outcomes. For example, is there a certain type of student who may perform better with a distance-learning platform? Many students with developmental disabilities and high behavioral needs have comorbid anxiety disorders. Perhaps these students would perform better in a more predictable and familiar environment with fewer distractions and social and academic demands. It is also possible that there may be differences in performance across ethnicity and socioeconomic or developmental levels. More work is needed to determine whether, and which, variables exist that may predict success with certain learning modalities over others.

In conclusion, Studies 1 and 2 lend support to the fact that this population requires continued service and that educators and clinicians must make data-based decisions that honor the ethical obligation to not abandon their clients and students even during global emergencies (Colombo et al., 2020). Although the NPS examined in the present study was forced to transition their service delivery model to a distance-learning program, the students continued to be offered education and service levels consistent with their IEP. Based on the data collected, it is possible that some form of education is more effective than eliminating services and education altogether. However, feasibility remains a consideration, as it was extremely difficult for students and their families to consistently access the program and their services, and many students were unable to log on. If in-home applied behavior analysis services are medically necessary and their personnel essential (Colombo et al., 2020; LeBlanc et al., 2020), what about applied behavior analysis services delivered in a school setting? Are special educators also essential workers? What are the detrimental impacts of closing school on students with special needs and their families? And how should these variables be taken into account when deciding how and when to open schools for in-person instruction? Unfortunately, there are more questions than there are answers. However, as the nation grapples with the decisions that lay ahead, it is imperative to also consider the needs of those most severely impacted during these emergencies. These students are a unique population with extreme challenging behaviors that compromise their safety. During times of crisis, such as the COVID-19 pandemic, these behaviors are likely to increase (LeBlanc et al., 2020), therefore necessitating the need for continued service levels and instruction. The present study attempted to begin to analyze whether these services and instruction would be feasibly and effectively delivered through distance learning. Although more research is needed, it is promising that educators felt there was some educational benefit and that students who participated maintained or continued to make progress on a majority of their IEP goals. However, this is only a small first step, and more work is needed to fully understand distance learning with this population.
